# The role of auditory abilities in basic mechanisms of cognition in older adults

**DOI:** 10.3389/fnagi.2013.00059

**Published:** 2013-10-04

**Authors:** Massimo Grassi, Erika Borella

**Affiliations:** Department of General Psychology, University of PadovaPadova, Italy

**Keywords:** auditory abilities, aging, working memory, inhibition, processing speed

## Abstract

The aim of this study was to assess age-related differences between young and older adults in auditory abilities and to investigate the relationship between auditory abilities and basic mechanisms of cognition in older adults. Although there is a certain consensus that the participant’s sensitivity to the absolute intensity of sounds (such as that measured via pure tone audiometry) explains his/her cognitive performance, there is not yet much evidence that the participant’s auditory ability (i.e., the whole supra-threshold processing of sounds) explains his/her cognitive performance. Twenty-eight young adults (age <35), 26 young–old adults (65 í age í 75), and 28 old–old adults (age >75) were presented with a set of tasks estimating several auditory abilities (i.e., frequency discrimination, intensity discrimination, duration discrimination, timbre discrimination, gap detection, amplitude modulation detection, and the absolute threshold for a 1 kHz pure tone) and the participant’s working memory, cognitive inhibition, and processing speed. Results showed an age-related decline in both auditory and cognitive performance. Moreover, regression analyses showed that a subset of the auditory abilities (i.e., the ability to discriminate frequency, duration, timbre, and the ability to detect amplitude modulation) explained a significant part of the variance observed in the processing speed of older adults. Overall, the present results highlight the relationship between auditory abilities and basic mechanisms of cognition.

## INTRODUCTION

It is well known that hearing declines with age (Cruickshanks et al., 1998). For example, old listeners have higher absolute thresholds than young normal-hearing listeners, in particular at high frequencies (e.g., Mazelová et al., 2003). With aging, however, hearing changes in other, different ways (e.g., Pichora-Fuller and Singh, 2006). Older people’s ability to discriminate between audible sounds and their ability to detect specific sounds characteristics worsen. Here, we refer to the whole supra-threshold processing of sounds as “auditory abilities” (Kidd et al., 2007). It is well documented that auditory abilities deteriorate with age: older adult listeners have worse performances (i.e., higher difference limens) than young normal-hearing listeners in several auditory tasks such as frequency discrimination (He et al., 1998; Clinard et al., 2010; Fitzgibbons and Gordon-Salant, 2010), intensity discrimination (He et al., 1998), or duration discrimination (Fitzgibbons and Gordon-Salant, 1994, Fitzgibbons and Gordon-Salant, 1995; Humes, 2005). In addition, performances of older adults in comparison to young-normal adults have been observed to be worse in the temporal processing of sounds, i.e., in sinusoidal amplitude modulation (SAM) detection thresholds and gap detection thresholds (Takahashi and Bacon, 1992; He et al., 1999; Humes et al., 2009,^2010^; Kumur and Sangamanatha, 2011). Note that performances of older adult listeners are not systematically worse than those of young-normal listeners. For example, the ability to discriminate synthetic timbres (i.e., spectral discrimination; Shrivastav et al., 2006) does not seem to decline with age.

With aging, other abilities show a decline, in particular, those that rely on attentional resources (e.g., Park et al., 2002; Borella et al., 2011). Because some of these abilities are correlated, Verhaeghen and Salthouse (1997) hypothesized a limited number of mechanisms, the so-called basic mechanisms of cognition. Basic mechanisms of cognition are, for example, working memory, processing speed, and cognitive inhibition, and are known both to modulate performance in various cognitive domains related to everyday activities (e.g., reading comprehension and problem solving; Borella et al., 2011) and to decline with age (de Ribaupierre, 2001; Park et al., 2002; Craik and Salthouse, 2008).

**FIGURE 1:**
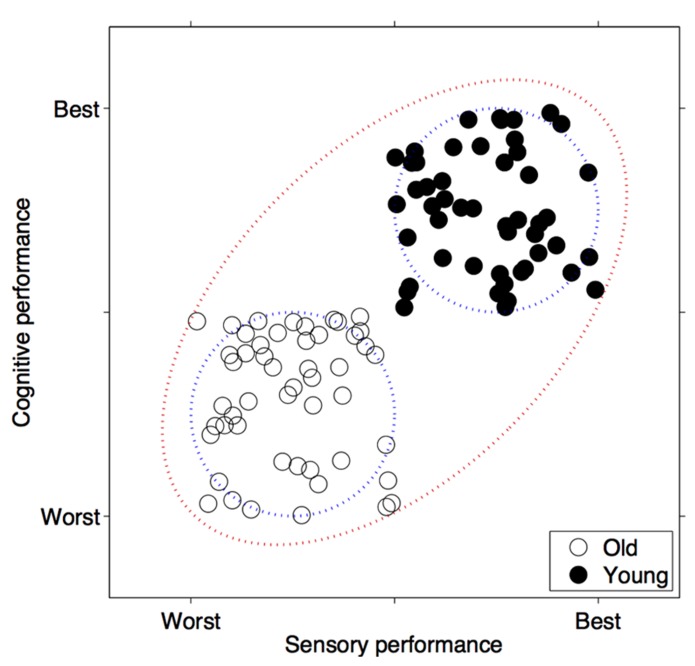
**Statistical artifact when correlating sensation and cognition by including the extremes of the age continuum.** The graph depicts two hypothetical age groups of subjects characterized by, respectively, poor sensory and poor cognitive performance (old) and good sensory and good cognitive performance (young). In the data represented, the correlation between sensory and cognitive performance (blue circles) is null within both groups: respectively *r*(old) = 0.004 and *r*(young) = -0.062. In contrast, regarding the correlation between sensory and cognitive performance calculated including all participants (red circle), the correlation is strong: *r*(all) = 0.719. The same artifact affects regression analysis.

For a long time authors have hypothesized a relationship between sensory acuity and cognitive faculties (e.g., Galton, 1883). More recently, Lindenberger and Baltes hypothesized that sensory aging causes cognitive aging (Lindenberger and Baltes, 1994; Baltes and Lindenberger, 1997). After Lindenberger and Baltes, other types of relationships have been suggested (see Schneider and Pichora-Fuller, 2000 for a detailed overview). The exact form of the relationship is still unknown. However, the relationship, for example, between audition and cognition, has been observed in a number of studies with respect to the absolute threshold of the participant (Valentijn et al., 2005; Tay et al., 2006; Lin et al., 2011) as well as the auditory abilities subtending the temporal processing of auditory stimuli (e.g., Humes, 2005; Humes et al., 2009,^2010^). The strength of such a relationship is still a matter of debate. According to some authors, the strong correlation observed by some studies might be a statistical artifact (Hofer et al., 2003). Some of the studies often correlated sensation and cognition by including the extremes of the age continuum (e.g., participants below 30 years of age vs. participants older than 75 years of age). However, since both cognitive and sensory performance decline with age, the correlation might be boosted due to these large differences between the groups. In other words, if authors correlate the data by including the performance of very different age groups they might observe a correlation between cognitive and sensory performance, although this correlation is null within each age group. When the correlational analyses are run on the data of older participants only the correlation between cognition and sensation is weak and in some case null (e.g., Hofer et al., 2003; Humes et al., 2009,^2010^). The same artifact affects other types of statistics such as regression analysis. **Figure 1** illustrates an example of this statistical artifact.

Several aspects concerning both the cognitive side and the auditory side have yet to be investigated. Periodic sounds (e.g., speech-vowels, musical tones) differ along four main dimensions: intensity, frequency, duration, and spectral shape. The ability of one listener to discriminate sounds along these dimensions is thought to explain the listener’s ability to discriminate the sounds in the environment (Moore, 2003). In young listeners, the ability to discriminate sounds along the above dimensions correlates with the listener’s intelligence (Watson, 1991). However, to the best of our knowledge, only the relationship between duration discrimination and measures of verbal, non-verbal, and total IQ (Humes, 2005) has been investigated in older adults. On the cognitive side, the cognitive abilities have been often assessed by means of a subset of the Wechsler Adult Intelligence Scale (WAIS) test (Humes, 2005; Humes et al., 2009,^2010^). However, this test is not specifically devoted to assess either working memory or cognitive inhibition.

The present study investigated age-related differences between young (age <30), young–old (65 ≤ age ≤ 75) and old-old adults (age >75) in auditory abilities spanning from temporal abilities (i.e., gap detection, SAM detection, and duration discrimination) and extending to the ability to discriminate frequency, intensity, and spectral shape. In addition, the study investigated the relationship between the above auditory abilities and working memory, cognitive inhibition, and processing speed. In line with the literature, we expected (and observed) an age-related decline of auditory and cognitive abilities. In addition, we observed that a subset of auditory abilities accounted for a significant part of the variance of the cognitive abilities of older adults. Note that here, in line with Humes et al. (2009), the relationship between audition and cognition has been investigated by analyzing the data of the older participants only.

## MATERIALS AND METHODS

### PARTICIPANTS

Thirty young adults (18–34 years of age), 60 older adults (64–88 years of age) were recruited for the study. The group of older adults was split into two groups: participants with age ranging from 64 to 75 (young–old, *n* = 30) and participants older than 75 (old–old, *n* = 30), because of the more pronounced cognitive and sensory decline in late adulthood (Baltes and Mayer, 1999). Participants were all Italian native speakers and volunteered for the study. They were community dwellers and were recruited by word of mouth. All participants that either reported hearing problems, wearing hearing aids, or that fit the “exclusion criteria” proposed by Crook et al. (1986) – i.e., history of head trauma; any neurological or psychiatric illness; history of brain fever; dementia or any other state of consciousness alteration; use of benzodiazepines in the previous three months; use of illicit drugs; visual, auditory and/or motor impairment; any symptomatic cardiovascular condition, breathing problems, or pathologies causing possible cognitive impairments – were excluded from the study. Older adults were selected on the basis of a physical and health questionnaire. Moreover, only older adults with a Mini Mental State score (MMSE; Folstein et al., 1975) over 27 were retained for the study. Because four young–old and two old–old participants had a MMSE of 24 or lower, they were excluded from the study. In addition, two of the young participants did not complete all the auditory tests and were therefore excluded from the study. The final sample was thus composed of 28 young, 26 young–old, and 28 old–old participants. Young and older adults did not differ on either the vocabulary test (Wechsler, 1981), *F* < 1, or for educational level, *F*(2,81) = 1.12, *p* = 0.33, ŋ_p_^2^ = 0.03. These results are similar to the literature that shows a substantial maintenance in vocabulary skills in normal aging (e.g., Park et al., 2002). Moreover, all participants performed above the cut-off for their age and education in the working memory test, and in an indirect measure of attentional control – intrusion errors – provided by this test (see Borella et al., 2008; De Beni et al., 2008). Participants’ background data are reported in **Table 1**.

**Table 1:** Characteristics of the participants by age group.

	Young (57% female)		Young–old (27% female)		Old–old (27% female)	
			
	*M*	SD	*M*	SD	*M*	SD
Age	25.29	5.30	68.00	2.47	81.04	3.87
Vocabulary	36.50	8.72	36.23	11.04	36.96	9.49
Education	13	0.00	12.92	0.27	12.96	0.19

### AUDITORY MEASURES: APPARATUS

Sounds were synthesized in real time at 44.1 kHz and 16 bits resolution, using MATLAB (©Mathworks) that was running on a Sony VAIO laptop that also controlled the experiment. The output of the sound card was presented diotically through Sennheiser HD 280 PRO headphones. The overall level for the sounds presented in the experiment was 50 dB above the participant’s absolute threshold for a 1 kHz pure tone (50 dB SL), i.e., about ~60–90 dB SPL depending on the participant. Tests were always run in quiet rooms (i.e., noise level at the participant’s ear below 35 dB A).

### AUDITORY MEASURES: STIMULI AND PROCEDURE

The experimental procedure described here was in accordance with the Declaration of Helsinki (Sixth revision, 2008). Participants completed seven tests: one absolute threshold measurement for a 1 kHz pure tone of 500 ms, followed by six classic psychoacoustical tests (see below for further details). First, the participant’s absolute threshold for a 1 kHz tone presented in isolation was estimated twice via the method of limits. In each trial the participant was presented with a tone and s/he was asked whether s/he heard the tone or not. If the answer was “yes” the tone’s level was attenuated by 5 dB and the attenuated tone was presented to the participant. This was repeated until the participant asserted s/he could no longer hear the tone. The absolute threshold was calculated as the mean between the level not heard and the last level heard by the participant. Such a measure enabled the experimenters to present the sounds of the successive psychoacoustical tests at the subjective level of 50 dB SL for all participants. After the 1 kHz absolute threshold, the participants ran six tests within a single listening session: (i) pure tone frequency discrimination, (ii) pure tone intensity discrimination, (iii) pure tone duration discrimination, (iv) gap detection, (v) SAM detection, and (vi) spectral shape discrimination. For each test, the participant performed two blocks of 20 trials. In each trial the participant was presented with three sound-intervals separated by a 500 ms silence. Two intervals were identical (the standards), whereas one interval (the variable) differed in one acoustical characteristic. For example, in the frequency discrimination test, the frequency of the variable interval was higher than that of the standards of a certain amount delta. After each trial, the participant was asked to report during which of the three observation intervals was the variable interval. The order of standards and variable was randomized before each trial. At the first trial of each block, delta was set in such a way to offer an easy trial to the participant. In the successive trials, delta was changed as a function of the participant’s response, according to a psychophysical adaptive procedure (maximum likelihood procedure; Green, 1990; Grassi and Soranzo, 2009) that tracked 72% of the participant’s psychometric function. At the first trial of each test’s block, participants were familiarized with the test by iteratively running the first trial of the block (the easiest) until she/he understood the task. Participants completed the six psychoacoustical tests in random order. The total duration of the 1 kHz absolute threshold measurement test and the six psychoacoustical tests was ~30 min.

#### Frequency, intensity, and duration discrimination

The standard intervals were two 1 kHz pure tones of 500 ms of duration gated on and off with two 10 ms raising cosine ramps. The variable interval was identical to the standard tone but had a higher frequency (or higher intensity, or longer duration). The frequency, the duration, and the intensity of the variable were let to home in on the participant’s discrimination threshold within a range of, respectively, 1000.1–1500 Hz, 500.1–900 ms, and 50.01–65 dB SL.

#### Spectral shape discrimination

The standard intervals were two, 500 ms long complex tones including the first five harmonics of a 333.3 Hz fundamental frequency and gated on and off with two 10 ms raising cosine ramps. All harmonics had identical amplitude and were added in phase so that the spectral centroid of the complex tone was 1 kHz. The variable interval was identical to the standard but had the third harmonic of a higher level than the remaining harmonics. This difference made the timbre of standards and variable different. In order to prevent participants’ responding by “hearing out” the intensity of the augmented harmonic, the overall level of the standards and the variable was randomized in each trial within the range of 5 dB. The level of the third harmonic of the variable interval was let to home in on the participant’s discrimination threshold within a range of 0.1–30 dB higher than the level of the other harmonics.

#### Gap detection and SAM detection

The standard intervals were two identical 500 ms long Gaussian noises gated on and off with two 10 ms raising cosine ramps. In gap detection, the variable interval was identical to the standard but its temporal center was cleared to create the gap. The gap was gated on and off with two 0.5 ms raising cosine ramps. The duration of the gap was let to home in on the participant’s detection threshold within a range of 0.1–64 ms. In SAM detection, the variable interval was identical to the standard but was amplitude modulated by a 10 Hz sinusoidal modulator. The modulation depth was let to home in on the participant’s threshold within a range of -60 to 0 dB (i.e., no modulation).

### COGNITIVE MEASURES: WORKING MEMORY AND COGNITIVE INHIBITION

Working memory capacity was evaluated with the Italian version of the listening span test (Borella et al., 2008; adapted from Daneman and Carpenter, 1980). The task consisted of an increasing number of two, three, four, five, six sequences of simple sentences presented via audio CD. The sentences varied between 6 and 12 words in length and the last word of the sentences could be composed of two, three, four, or five syllables. Participants were instructed to listen to each sentence, judge its plausibility (state whether it was true or false), and retain the last word. Half of the sentences were true and half were false. At the end of each sequence, participants were required to recall all the final words, following the correct order of presentation. Each participant adjusted the presentation level of the test to a comfortable listening level. The test was preceded by two simple sentences that were used by the experimenter to assess whether the sound level was adequate to perform the task. The total number of final words correctly recalled in the correct order (max = 40) during the whole test represented a measure of the participant’s working memory capacity. To ensure that the participants were not trading off between processing the sentences and remembering the words, an 85% accuracy criterion on the judgment task was required. None of the participants performed below this criterion.

In addition, the proportion of intrusion errors (words presented during the task that were recalled by the participant but that were not the last word of a sentence) was computed by dividing the total number of intrusions by the total number of correctly recalled words (Robert et al., 2009). This procedure assessed the ability to exhibit control over the permanence of information in working memory and is considered a measure of cognitive inhibition (e.g., Robert et al., 2009).

### COGNITIVE MEASURES: PROCESSING SPEED – LETTER COMPARISON TASK (ADAPTED FROM Salthouse and Babcock, 1991)

Participants were asked to decide whether two strings of letters were identical or not. String-pairs were written on two pages, each containing two columns of 21 string-pairs. Each string of a string-pair could be composed of three, six, or nine consonants and had no meaning (e.g., they were not identical to abbreviations and/or common acronyms). The participant had to respond as quickly as possible by writing “Sì” (“Yes” for identical) or “No” (“No” for different) along a line dividing the members of each string-pair. The experimenter measured the time to complete each page by means of a stopwatch. The dependent variable was the total time (in seconds) taken to complete the two pages.

### GENERAL PROCEDURE

The experiment was divided in two sessions that were performed by the participant within the same day. In the first session (cognitive), the MMSE was presented to older adults only. Then, each participant took the vocabulary test and the working memory test followed by the processing speed test. In the successive auditory session, participants took all the auditory tests. The whole duration of the experiment was ~90 min. Participants could take breaks at any time during the experiment.

### DATA ANALYSIS

To assess the age-related differences between young, young-old and old-old adults in cognitive and auditory performance we used a set of univariate ANOVAs with the age group (young vs. young–old vs. old–old) as a between factor variable. Successively, to assess the relationship among the auditory tests, we used a set of correlations and, furthermore, to account the role of age, correlations were recalculated by partialling out the age of the participant. We also calculated the correlations among the cognitive tests by partialling out (or not) the age of the participant. Then, a principal component analysis was conducted to reduce the number of auditory measures to a limited number of auditory factors. Finally, a set of hierarchic regressions was run to determine the extent to which auditory factors accounted for age-related variance in working memory, cognitive inhibition, and processing speed. Correlations, principal component analysis, and regressions were calculated by analyzing the data of the older participants only (i.e., all participants older than 65 years of age) because the performance of these two groups did not differ in the auditory tests whereas that between young adults and older adults did (see results of the Tukey *post hoc* test). For all the analyses, alpha value was set to 0.05.

## RESULTS

### AGE-DIFFERENCES AMONG AGE GROUPS: AUDITORY MEASURES

**FIGURE 2:**
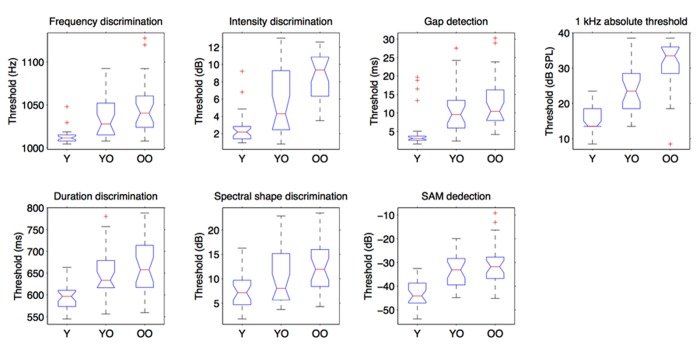
**Performance of the three age-groups in the auditory tasks.** “Y” are young participants (age <35), “YO” are young–old participants (65 ≤ age ≤ 75) and “OO” are old–old participants (age >75).

The main effect of age groups was significant for all the auditory measures (1 kHz absolute threshold, *F*(2,79) = 62.13, *p* < 0.001, ŋ_p_^2^ = 0.61; frequency discrimination: *F*(2,79) = 15.19, *p* < 0.001, ŋ_p_^2^ = 0.28; intensity discrimination, *F*(2,79) = 29.01, *p* < 0.001, ŋ_p_^2^ = 0.42; spectral shape discrimination, *F*(2,79) = 5.92, *p* < 0.01, ŋ_p_^2^ = 0.13; duration discrimination, *F*(2,79) = 14.55, *p* < 0.001, ŋ_p_^2^ = 0.27; gap detection, *F*(2,79) = 11.86, *p* < 0.001, ŋ_p_^2^ = 0.23; and SAM detection, *F*(2,79) = 22.43, *p* < 0.001, ŋ_p_^2^ = 0.36). Tukey *post* *hoc* test revealed that young adults had lower thresholds than young–old and old–old adults (*p* < 0.001 for all comparison), which did not differ significantly from each other, in all these auditory measures. The only exception was the spectral shape task. Here, old–old adults had higher thresholds than young adults (*p* < 0.001) but young–old adults did not differ significantly from both young and old–old adults (see **Figure 2**).

### AGE-DIFFERENCES AMONG AGE GROUPS: COGNITIVE MEASURES

**FIGURE 3:**
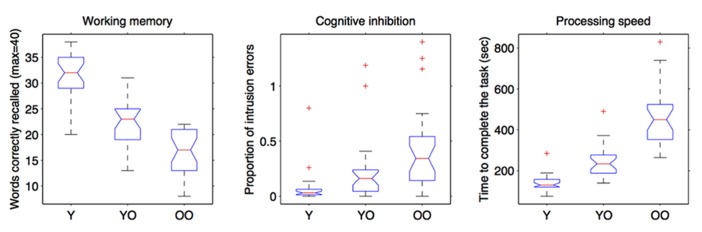
**Performance of the three age-groups in working memory, cognitive inhibition, and processing speed tasks.** “Y” are young participants (age <35), “YO” are young–old participants (65 ≤ age ≤ 75) and “OO” are old–old participants (age >75).

For the working memory test, the main effect of age group was significant, *F*(2,79) = 79.24, *p* < 0.001, ŋ_p_^2^ = 0.67: young adults recalled more correct words than old–old adults (*p* < 0.001) and young–old adults (*p* < 0.001). The latter two groups did not differ from each other (see **Figure 3**). With respect to cognitive inhibition, we observed the significant main effect of age group, *F*(2,79) = 10.31, *p* < 0.001, ŋa_p_^2^ = 0.21: young–adults and young–old adults did not differ from each other in the number of intrusions whereas old–old adults produced a significantly higher number of intrusions than both the younger adult groups (*p* < 0.001 and *p* < 0.05, respectively, see **Figure 3**). For the processing speed, the significant main effect of age group, *F*(2,79) = 79.78, *p* < 0.001, ŋ_p_^2^ = 0.67, showed that young–old and old–old adults were slower than young adults (for both, *p* < 0.001). Moreover, old–old adults were also significantly slower than young–old adults (*p* < 0.001, see **Figure 3**).

### CORRELATIONS AND MULTIVARIATE ANALYSES

#### Auditory measures

Overall, small correlations were found (see **Table 2**). In particular, age correlated positively with spectral shape discrimination, intensity discrimination, and the absolute threshold for the 1 kHz tone. Moreover, SAM detection was correlated positively with duration discrimination, frequency discrimination, and gap detection. Finally, frequency discrimination correlated positively with duration discrimination, intensity discrimination, and spectral shape discrimination. The 1 kHz absolute threshold correlated with the frequency discrimination, the spectral shape discrimination and, above all, with intensity discrimination. The correlations between the psychoacoustical tests did not vary when controlling for age, except for those between frequency discrimination and intensity discrimination and those between SAM detection and gap detection that became non-significant. Also the correlation between absolute threshold and frequency discrimination, and that between absolute threshold and spectral shape discrimination became non-significant. This suggests that the above relationships were possibly mediated by age.

**Table 2:** Correlation matrix between age and the auditory measures.

	Age	1	2	3	4	5	6	7
Frequency discrimination (1)	0.19	–	0.39^**^	0.22	0.05	0.28	0.38^*^	0.20^*^
Duration discrimination (2)	0.08	0.40^**^	–	0.13	0.03	0.35^*^	0.24	0.22
Intensity discrimination (3)	0.39^**^	0.27^*^	0.15	–	0.06	-0.10	0.08	0.53^**^
Gap detection (4)	0.03	0.05	0.03	0.06	–	0.27*	-0.04	-0.10
SAM detection (5)	0.14	0.30^*^	0.36^**^	-0.04	0.27	–	0.19	0.16
Spectral shape discrimination (6)	0.29^*^	0.42^**^	0.25	0.18	-0.03	0.22	–	0.19
1-kHz absolute threshold (7)	0.52^**^	0.28^*^	0.23	0.62^**^	-0.07	0.21	0.31^*^

*Raw correlations (*n* = 54) are presented below the diagonal; correlations above the diagonal are controlled for age.*

* *p* < 0.05;

** *p* < 0.01.

#### Cognitive measures

Age correlated negatively with working memory performance [*r*(54) = -0.66, *p* < 0.001], and positively with cognitive inhibition [i.e., higher number of intrusions *r*(54) = 0.39, *p* = 0.003] and processing speed [i.e., longer completion times, *r*(54) = -0.73, *p* < 0.001]. In addition, participants who demonstrated better working memory performance were also those with faster processing speed [*r*(54) = -0.55, *p* < 0.001] and with more efficient inhibitory mechanisms [*r*(54) = -0.67, *p* < 0.001]. Finally, processing speed correlated positively with cognitive inhibition [*r*(54) = 0.28, *p* = 0.03]. Two of the three correlations between the cognitive tests varied when controlling for age, namely, that between inhibition and processing speed [*r*(54) = 0.00, *p* > 0.05] and that between processing speed and working memory [*r*(54) = -0.11, *p* > 0.05]. In contrast, the correlation between inhibition and working memory did not vary: *r*(54) = -0.60, *p* < 0.001.

### PRINCIPAL COMPONENT ANALYSIS

Before assessing the relationship between auditory and cognitive measures, the seven auditory measures were subjected to an exploratory principal component analysis with direct varimax rotation. This was done to examine whether performance in the different auditory tests reflected a single ability or a disparate set of abilities. The cognitive measures were not subjected to the principal components analysis in order to assess the role of auditory abilities on each of the basic mechanisms of cognition (i.e., working memory, cognitive inhibition, and processing speed). Results showed a three factors solution that had eigenvalues greater than 1.0. Loadings higher than 0.6 were used to interpret the factors. The rotated pattern matrix is shown in **Table 3**. Frequency discrimination, duration discrimination, spectral shape discrimination, and SAM detection loaded on Factor 1. The 1 kHz absolute threshold and the intensity discrimination loaded on Factor 2 (none of these measures had loadings of greater than 0.6 on Factor 1 and Factor 2). Gap detection alone loaded Factor 3. The Factor 1 explained 34% of the variance, while Factor 2 and Factor 3 explained, respectively, 19% and the 15% of the variance (see **Table 3**). Factor scores resulting from this three-factors solution were generated and saved for a linear regression analysis. The correlations between the three factors were not significant. The results returned by the principal component analysis are consistent with the results of the correlation analyses. Furthermore, the lack of correlation between Factor 1, 2, and 3 stresses the orthogonally and independency of the three factors.

**Table 3:** Rotated factor matrix of the principal component analysis.

Measures	Factor 1	Factor 2	Factor 3
Frequency discrimination	0.73	0.23	0.03
Duration discrimination	0.72	0.05	0.08
Spectral shape discrimination	0.67	0.21	-0.17
SAM detection	0.61	-0.09	0.53
Intensity discrimination	0.05	0.93	0.04
1-kHz absolute threshold	0.29	0.82	-0.03
Gap detection	-0.06	0.04	0.93

### EFFECTS OF AUDITORY MEASURES ON AGE-RELATED VARIANCE IN COGNITIVE ABILITIES

Hierarchical multiple regression analyses were run. Two models were evaluated. In Model 1, age was entered as a predictor to determine the amount of variance in, respectively, working memory, cognitive inhibition, and processing speed. Model 2 extended Model 1 and examined whether the Factor 1, 2, or 3 significantly reduced the contribution of age in explaining variance of, respectively, working memory, cognitive inhibition, and processing speed. The net contribution of the auditory factors was calculated as follows:

$$100\times [(R_{age}^{2}-R_{change}^{2})/R_{age}^{2}],$$

where R^2^_age_ is the variance accounted for by age (i.e., Model 1) and R^2^_change_ is the change in *R*^2^ after the introduction of the auditory factors (see Baudouin et al., 2009 for a similar analysis).

Model 1 showed that age predicted 44% of the variance in working memory, 14% in cognitive inhibition, and 53% in processing speed. Model 2 revealed that auditory factors explained 27% of the variance in working memory and 39% in processing speed. In contrast, auditory factors did not explain the variance of cognitive inhibition, and age remained the only significant predictor. Interestingly, only Factor 1 (i.e., that including frequency, duration, spectral shape discrimination, and SAM detection) made a significant contribution in processing speed (β = 0.39, *p* < 0.001). However, this contribution was small: age still explained 45% of the variance.

## DISCUSSION

The current study investigated age-related differences between young, young–old and old–old adults in auditory abilities and cognitive abilities. In addition, it investigated whether auditory abilities could explain the cognitive performance of older adults. Overall, the results confirm the literature findings. The two groups of older adults performed worse than young adults in all auditory tasks with the exception of spectral shape discrimination test where performance of young–old was limitedly preserved (e.g., Shrivastav et al., 2006). Moreover, the two groups of older adults performed worse than young adults in all cognitive tasks with the exception of cognitive inhibition where performance of young–old was also preserved (e.g., Carretti et al., 2012). In summary, the results of the current study, together with those of its predecessors, reveal that auditory and cognitive abilities decline with aging.

Concerning the auditory side, results showed that many of the tasks investigated were correlated. Moreover, the majority of the correlations between auditory measures were partially mediated by age. The successive principal component analysis grouped auditory abilities in three distinct factors that mirrored largely the results of the correlations, suggesting that the auditory measures investigated here might represent different constructs that are differently sensitive to aging. We discuss these three factors starting from the last. Factor 3 coincides with gap detection alone. This factor did not correlate with others, nor did it contribute to explaining part of the cognitive performance in older participants. This result is partially in contrast with the finding of Humes et al. (2010) that observed that the performance in the gap detection task could account for a significant part of the variance in the cognitive performance. However, (i) in that study the contribution of the gap detection task in explaining the cognitive performance was very limited (i.e., 10%); (ii) that study had a much larger statistical power than the current study, thus, the chances to observe statistically significant results were higher. In any case, it is interesting to note that here, the ability to detect temporal gaps did not saturate together with SAM detection and/or duration discrimination, which are also considered measures of temporal auditory processing (see Humes, 2005 for duration discrimination). Factor 2 includes tasks that manipulate the overall intensity of a tone, i.e., the 1 kHz absolute threshold as well as the participant’s ability to discriminate between tones’ intensities. This factor also did not contribute to explaining the individual differences in the cognitive abilities of older adults. Note that the absolute threshold is often found in a relationship with the cognitive abilities (e.g., Valentijn et al., 2005; Tay et al., 2006; Lin et al., 2011). However, some studies did not observe such a relationship (e.g., Hofer et al., 2003; Humes et al., 2009). Perhaps, the reason why some authors observe a relationship whereas others do not depends on the way the correlational analyses are carried out: i.e., whether authors perform (or do not perform) analyses by including the data of participants of very different ages (e.g., Hofer et al., 2003; Humes et al., 2009). Finally, Factor 1 explained part of the variance of cognitive abilities of older adults, in particular processing speed. This suggests that auditory abilities, and in particular the ability to discriminate sounds by frequency, duration, long-term spectrum, and envelope explains the time needed to conduct central/computational operations. It is not simple to interpret Factor 1. However, some of the abilities included in Factor 1 play an important role in the capacity of a listener to understand speech (i.e., SAM detection and spectral shape discrimination; Takahashi and Bacon, 1992; Shrivastav et al., 2006) or to judge the prosody and the emotional content of speech (frequency discrimination; Lieberman and Michaels, 1962). Moreover, some of these abilities correlate with intelligence and academic aptitude in young listeners (Watson, 1991). In any case, the amount of variance explained by Factor 1 was small (i.e., 39%). As far as working memory and cognitive inhibition is concerned, here no auditory factor could explain any of these cognitive measures. Processing speed is a lower level mechanism in comparison to working memory and cognitive inhibition. Processing speed is related to the rate at which elementary cognitive operations are carried out and it is also a major factor contributing to age-related differences in cognition both in early and late development. Different studies have shown that processing speed accounts for a substantial part of the age-related variance in working memory (e.g., Salthouse and Babcock, 1991). It is possible that simple auditory abilities (such as those investigated in the current study) have a higher likelihood of explaining simpler cognitive mechanisms, such as processing speed, rather that complex ones such as working memory and cognitive inhibition. In addition, it is possible that the processing speed measure differentiated better the performances of the older participants in comparison to the measures of working memory and cognitive inhibition. The scores of older adults in the cognitive inhibition and the working memory tests were rather compressed. A compression in these scores could hide the possible relationships among cognitive inhibition, working memory, and the auditory tests.

Despite these results, the limitations of the present study have to be acknowledged. Firstly, it would be of interest to investigate a larger sample size to replicate the present results. Secondly, it would be of interest to carry out an adult life-span study, to clarify the relationship between age, auditory abilities, and cognitive abilities. In addition, it would be of interest to use non-auditory measures of cognitive abilities (here we used an auditory measure of working memory) in order to be able to understand whether the relationship between auditory abilities and cognitive abilities is (or is not) sense-dependent. Finally, it would be interesting to extend cognitive abilities to other age-dependent abilities such as attention.

In conclusion, it is long-believed that there is a relationship between cognition and sensory performance (e.g., Galton, 1883).Therefore, when sensory performance worsens (i.e., in aging), cognitive performance should also worsen, yet empirical research (including the current) often observes a moderate to weak relationship between sensory acuity and cognition.

## Conflict of Interest Statement

The authors declare that the research was conducted in the absence of any commercial or financial relationships that could be construed as a potential conflict of interest.
